# Response to the Letter: Mean Platelet Volume to Platelet Count Value May Not Be a Prognostic Marker in Patients with Crimean–Congo Hemorrhagic Fever

**DOI:** 10.5041/RMMJ.10421

**Published:** 2020-10-14

**Authors:** Yusuf Kenan Tekin, Aynur Engin

**Affiliations:** 1 Department of Emergency Medicine, Sivas Cumhuriyet University Medical Faculty, Sivas, Turkey; 2 Department of Infectious Diseases and Clinical Microbiology, Sivas Cumhuriyet University Medical Faculty, Sivas, Turkey

## TO THE EDITOR

We carefully read the comments of our respected colleagues Cengiz Beyan and Esin Beyan regarding our article, “An Evaluation of the Different Serum Markers Associated with Mortality in Crimean–Congo Hemorrhagic Fever.”^1^

We actually investigated many blood parameters in patients with Crimean–Congo hemorrhagic fever (CCHF), including white blood cell counts, hemoglobin, platelet counts, mean platelet volume (MPV), neutrophils, lymphocytes, red cell distribution width (RDW), and high-sensitivity C-reactive protein (hs-CRP). Statistical analysis was conducted by our colleagues who are experts in this field. Statistical analysis revealed a statistically significant difference in the mean values of white blood cell, neutrophil, lymphocyte, hs-CRP, MPV, RDW, MPV-to-platelet count ratio (MPVPCR), and RDW-to-platelet count ratio (RDWPCR) in the survivors versus non-survivors.

Firstly, it should be noted that the device used to measure complete blood count in our university hospital biochemistry laboratory was a BC-6800 (Mindray, Shenzhen, China). Our hospital biochemistry laboratory confirmed, in writing, that this was only device used throughout the study period.

It is well-known that CCHF can cause hospital-acquired infection with high mortality. In addition, our medical center has followed a large number of CCHF patients for several years. Thus, patients have been closely followed, and CCHF blood tests are sent to the laboratory as soon as possible.

Secondly, Beyan and Beyan commented that MPV cannot be used as a prognostic marker in acquired diseases. However, a literature search reveals a number of articles reporting MPV as a useful prognostic marker in some diseases. For example:

- Vardon-Bounes et al.^2^ reported that MPV was an independent predictive factor of 90-day mortality. They suggested that continuous monitoring of MPV may be a useful parameter to stratify mortality risk in septic shock.
- Ma et al.^3^ reported that high MPV can be considered as an independent biomarker for predicting 3-month mortality in patients with hepatitis B virus (HBV)-related decompensated cirrhosis.
- Lee et al.^4^ indicated that MPV measurements may be used as a prognostic marker of mortality in intensive care unit patients with pneumonia.

In addition, there are many articles in the literature reporting the relationship between MPVPCR and infection. Some of these are:

- Han et al.^5^ reported that MPVPCR ratio in pediatric patients with infectious mononucleosis was significantly higher compared with those in the controls. In receiver operating characteristic (ROC) analysis, they obtained a cut-off value of 3.42 for MPVPCR ratio with a sensitivity of 83.7% and a specificity of 76.0% for the prediction of infectious mononucleosis in pediatric patients.
- Djordjevic et al.^6^ investigated various biomarkers regarding the outcome in critically ill patients with secondary sepsis and/or trauma. Their study enrolled patients with peritonitis, pancreatitis, trauma with sepsis, and trauma without sepsis. They found that MPVPCR was significantly higher in non-survivors. In addition, patients with a Gram-positive blood culture had a significantly lower MPVPCR compared to patients with Gram-negative and polymicrobial blood cultures. When they compared polymicrobial and negative blood cultures, they found significantly higher MPVPCR values in patients with polymicrobial blood cultures. Djordjevic et al. reported that MPV was a very good independent predictor of lethal outcome.

Thirdly, Beyan and Beyan mentioned that MPV cannot be used as a diagnostic marker in acquired diseases. Actually, we do not recommend MPV as being useful for CCHF diagnosis, since its diagnosis is made using specific tests such as polymerase chain reaction (PCR) and enzyme-linked immunosorbent assay (ELISA). We suggested that MPVPCR levels may be a useful marker for the *prediction of mortality* in CCHF disease according to the statistical analysis of our study.

Finally, Beyan and Beyan criticized the absence of a healthy control group in our study. We stated in the manuscript that one of the limitations of our study was its retrospective nature. Furthermore, we advised that additional prospective studies are needed to confirm the suitability of MPVPCR as a mortality predictor in CCHF in the article.

Finally, our study was evaluated by referees who are experts in this field, and who found our study to be scientifically appropriate for publication.

For all the reasons explained above, we respectfully disagree with the opinions of our colleagues Cengiz Beyan and Esin Beyan.

## Abbreviations

CCHF: Crimean Congo hemorrhagic fever
hs-CRP: high-sensitivity C-reactive protein
MPV: mean platelet volume
MPVPCR: mean platelet volume-to-platelet count ratio
RDWPCR: red cell distribution width-to-platelet count ratio
